# Crystal structure of *N*-(4-oxo-2-sulfanyl­idene-1,3-thia­zolidin-3-yl)-2-(thio­phen-3-yl)acetamide

**DOI:** 10.1107/S2056989017007629

**Published:** 2017-05-31

**Authors:** Trung Vu Quoc, Linh Nguyen Ngoc, Cong Nguyen Tien, Chien Thang Pham, Luc Van Meervelt

**Affiliations:** a Faculty of Chemistry, Hanoi National University of Education, 136 Xuan Thuy, Cau Giay, Hanoi, Vietnam; b Faculty of Chemistry, Ho Chi Minh City University of Education, 280 An Duong Vuong Street, District No. 5, Ho Chi Minh City, Vietnam; c Department of Chemistry, Hanoi University of Science, 19 Le Thanh Tong Street, Hoan Kiem District, Hanoi, Vietnam; d Department of Chemistry, KU Leuven, Biomolecular Architecture, Celestijnenlaan 200F, Leuven (Heverlee), B-3001, Belgium

**Keywords:** crystal structure, thia­zolidine, thio­phene, rhodanine, polythio­phene

## Abstract

The title compound, C_9_H_8_N_2_O_2_S_3_, crystallizes with two mol­ecules (*A* and *B*) in the asymmetric unit. Both have similar conformations (overlay r.m.s. deviation = 0.209 Å) and are linked by an N—H⋯O hydrogen bond. In both mol­ecules, the thio­phene rings show orientational disorder, with occupancy factors of 0.6727 (17) and 0.3273 (17) for mol­ecule *A*, and 0.7916 (19) and 0.2084 (19) for mol­ecule *B*. The five-membered rings make an angle of 79.7 (2)° in mol­ecule *A* and an angle of 66.8 (2)° in mol­ecule *B*. In the crystal, chains of mol­ecules running along the *a*-axis direction are linked by N—H⋯O hydrogen bonds. The inter­action of adjacent chains through N—H⋯O hydrogen bonds leads to two types of ring structures containing four mol­ecules and described by the graph-set motifs *R*_4_^4^(18) and *R*_4_^2^(14).

## Abstract 2

The synthesis and crystal structure of a new thio­phene monomer containing an additional rhodanine heterocycle are reported. The crystal packing is sustained by N—H⋯O, C—H⋯O, C—H⋯S and C—H⋯π inter­actions.

## Chemical context   

Thio­phene, C_4_H_4_S, belongs to a class of aromatic five-membered heterocycles containing one S heteroatom. Thio­phene and its derivatives occur in petroleum or coal (Orr & White, 1990 ▸). Thio­phene-based compounds have applications in modern drug design (Santagati *et al.*, 1994 ▸), electronic and optoelectronic devices (Barbarella *et al.*, 2005 ▸), and conductive and electroluminescent polymers (Friend *et al.*, 1999 ▸). Also, several reviews of various aspects of thio­phene coordination and reactivity in transition-metal complexes have been reported (Barbarella *et al.*, 2005 ▸).
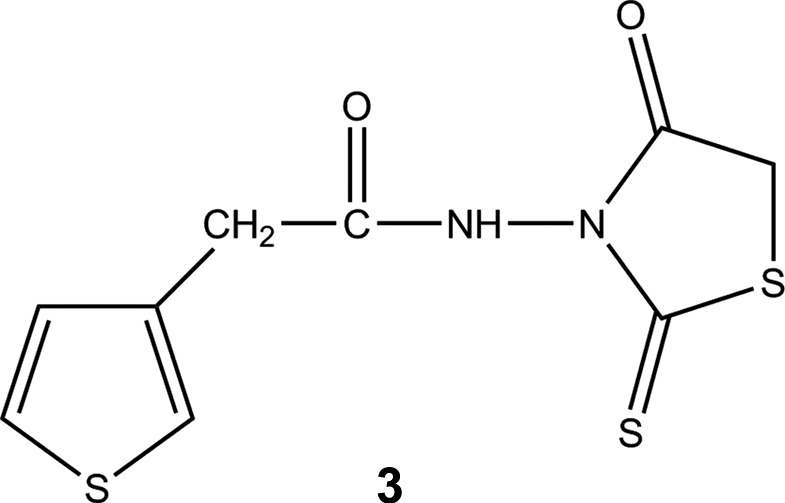


Derivatives of rhodanine (or 2-thioxo-1,3-thia­zolidin-4-one) have inter­esting pharmacological properties, such as the drug Epalrestat, which is an aldose reductase inhibitor used to treat diabetic neuropathy (Tomašić & Mašič, 2012 ▸). Some other rhodanine derivatives were designed and synthesized for detecting tau pathology in the brains of patients with Alzheimer’s disease (Ono *et al.*, 2011 ▸).

As a continuation of our research (Nguyen *et al.*, 2016 ▸; Vu *et al.*, 2016 ▸) on the chemical, physical and biological properties of new polythio­phenes, a new thio­phene monomer containing rhodanine has been prepared. In the presence of FeCl_3_, thio­phene monomers can be polymerized by C—C bond formation between the 2- and 5-positions of two subsequent thio­phene monomers, resulting in an extended π-conjugated system. We present here the synthesis and crystal structure of *N*-(4-oxo-2-sulfanyl­idene-1,3-thia­zolidin-3-yl)-2-(thio­phen-3-yl)acetamide, **3** [Chem scheme1].

## Structural commentary   

**Figure 1:**
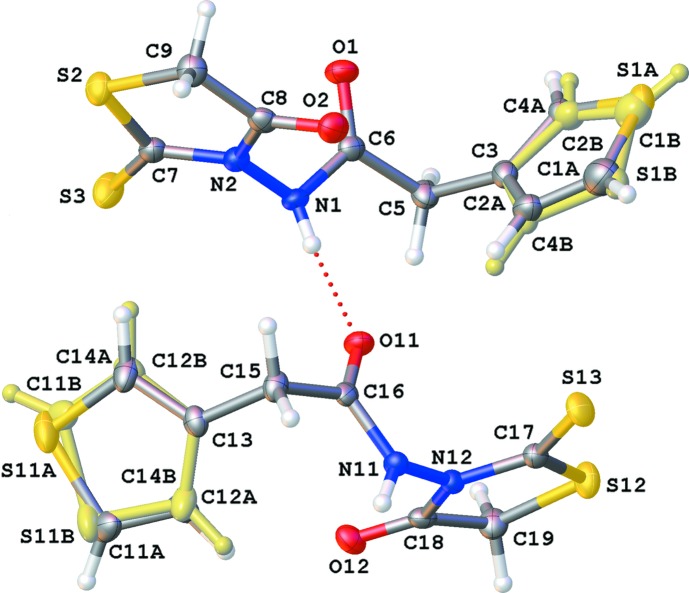
View of the asymmetric unit of the title compound, showing the atom-labelling scheme. Displacement ellipsoids are drawn at the 50% probability level. H atoms are shown as small circles of arbitrary radii. The minor component of the disordered thio­phene rings is shown in pale yellow.

Crystals of the title compound belong to the triclinic space group *P* 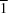 with two independent mol­ecules (*A* and *B*) per asymmetric unit (Fig. 1 ▸). In both mol­ecules, the thio­phene ring is disordered over two positions by a rotation of approximately 180° around the C5—C3 or C15—C13 bond for mol­ecules *A* and *B*, respectively [occupancy factors = 0.6727 (17) and 0.3273 (17) for mol­ecule *A*, and 0.7916 (19) and 0.2084 (19) for mol­ecule *B*]. In the current discussion, only the major components will be considered. The 1,3-thia­zolidine ring is almost planar (r.m.s. deviation = 0.020 Å for ring S2/N2/C7–C9 and 0.010 Å for ring S12/N12/C17–C19) with the N3-substitiuents N1 [0.141 (1) Å] and N11 [0.100 (1) Å] situated in the same plane (deviations from plane given in parenthesis). Both thio­phene rings are also planar as expected (r.m.s. deviation = 0.011 Å for ring S1*A*/C1*A*–C4*A* and 0.002 Å for ring S11*A*/C11*A*–C14*A*), with the substituents C5 [−0.065 (2) Å] and C15 [0.001 (1) Å] coplanar. In mol­ecule *A*, the heterocyclic rings make an angle of 79.7 (2)°; in mol­ecule *B*, this angle is 66.8 (2)°. Also, the amide group and the 1,3-thia­zolidine ring are oriented almost perpendicular to each other. In mol­ecule *A*, the plane through the atoms of the amide group (N1/C6/O1) makes an angle of 76.32 (8)° with the best plane through the 1,3-thia­zolidine ring; for mol­ecule *B*, this angle is 83.88 (6)°. Both mol­ecules in the asymmetric unit are linked by an N1—H1⋯O11 hydrogen bond (Table 1 ▸ and Fig. 1 ▸).

## Supra­molecular features   

**Figure 2:**
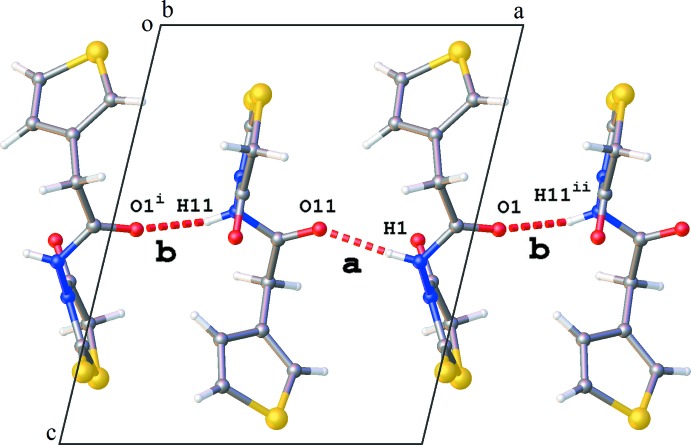
Part of the crystal packing of the title compound, showing a chain of mol­ecules along the *a* axis formed by N—H⋯O hydrogen-bond inter­actions **a** and **b** [see Table 1 ▸; symmetry codes: (i) *x* − 1, *y*, *z*; (ii) *x* + 1, *y*, *z*].

**Figure 3:**
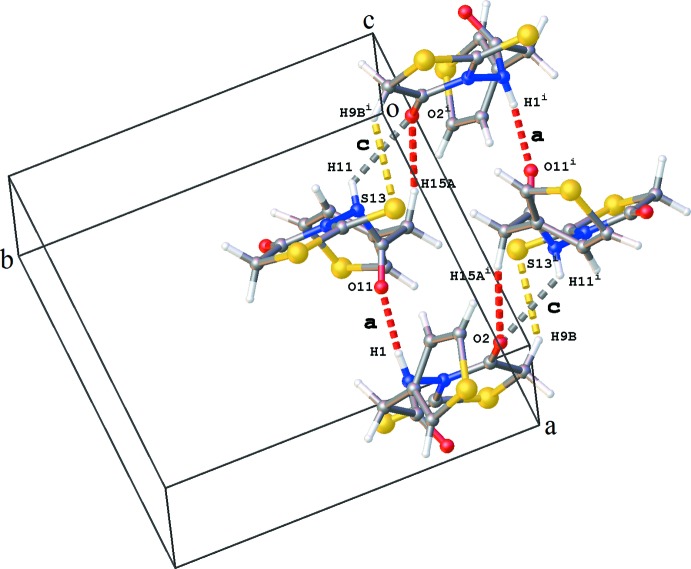
Ring of graph-set motif 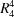
(18) formed by N—H⋯O hydrogen-bond inter­actions **a** and **c** [see Table 1 ▸; symmetry code: (i) −*x* + 1, −*y*, −*z* + 1].

**Figure 4:**
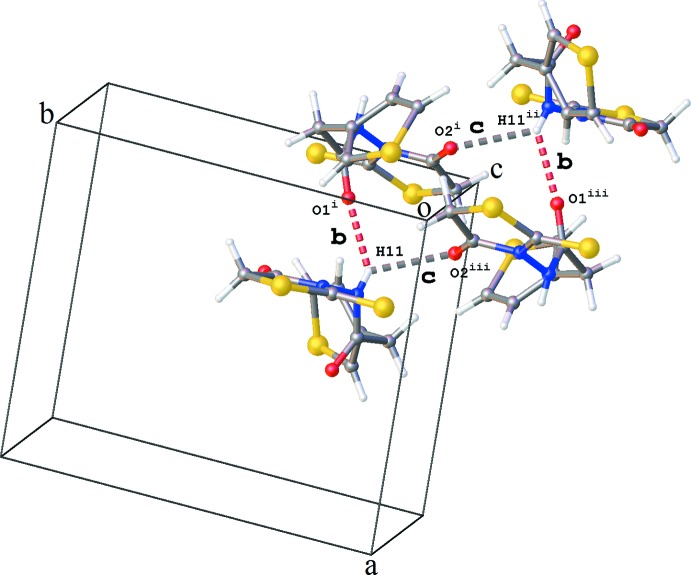
Ring of graph-set motif 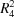
(14) formed by N—H⋯O hydrogen-bond inter­actions **b** and **c** [see Table 1 ▸; symmetry codes: (i) *x* − 1, *y*, *z*; (ii) −*x*, −*y*, −*z* + 1; (iii) −*x* + 1, −*y*, −*z* + 1].

The crystal packing is governed by hydrogen bonding. Chains of mol­ecules are formed along the *a*-axis direction by alternating N1—H1⋯O11 and N11—H11⋯O1 hydrogen bonds (Table 1 ▸ and Fig. 2 ▸). The inter­action of adjacent chains through N11—H11⋯O2 hydrogen bonds results in two different types of ring structures, each containing four mol­ecules: (i) a ring structure of graph-set motif 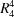
(18) showing also additional C—H⋯O and C—H⋯S inter­actions (Table 1 ▸ and Fig. 3 ▸), and (ii) a ring structure with graph-set motif 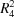
(14) (Fig. 4 ▸). The packing shows a number of additional C—H⋯O, C—H⋯S and weak C—H⋯π inter­actions (Table 1 ▸). The crystal packing contains no voids.

## Database survey   

A search of the Cambridge Structural Database (CSD, Version 5.38, last update February 2017; Groom *et al.*, 2016 ▸) for structures containing an *N*-substituted 2-thioxo-1,3-thia­zolidin-4-one ring gave 26 hits (169 hits when substituents at the 5-position are also allowed). In all cases, the 1,3-thia­zolidine ring can be considered to be planar, as the largest deviation from the best plane through the ring atoms was only 0.070 Å [for the complex bis­(rhodanine)copper(I) iodide; refcode VICJUM; Moers *et al.*, 1986 ▸]. The substituent at the N3 position is situated in the 1,3-thia­zolidine plane, with a largest deviation of 0.174 Å for the case with –NH_2_ as substituent (refcode EDEPUZ01; Jabeen *et al.*, 2007 ▸).

Rotational disorder in 3-CH_2_-thio­phene fragments is frequently observed (25 structures of the 67 fragments present in the CSD).

## Synthesis and crystallization   

**Figure 5:**
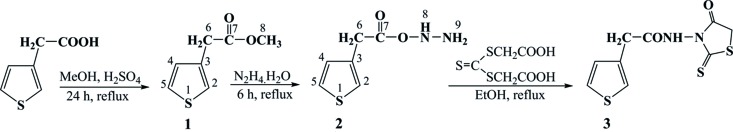
Reaction scheme for the title compound.

The reaction scheme to synthesize the title compound, **3**, is given in Fig. 5 ▸.

### Synthesis of methyl 2-(thio­phen-3-yl)acetate, 1   

Methyl thio­phene-2-acetate, **1** (5 mmol), was added to an excess of hydrazine hydrate (40 mmol) in ethanol (20 ml). The mixture was refluxed for 6 h. The reaction mixture was allowed to cool. The resulting precipitate was filtered and recrystallized from ethanol solution to give 0.57 g (yield 74%) of hydrazide **2** in the form of white crystals (m.p. 343 K). IR (Nicolet Impact 410 FTIR, KBr, cm^−1^): 3323, 3068 (ν_NH_), 3068, 2957 (ν_CH_), 1641 (ν_C=O_), 1526 (ν_C=C_ thio­phene). ^1^H NMR [Bruker XL-500, 500 MHz, *d*_6_-DMSO, δ (ppm), *J* (Hz)]: 7.22 (*dd*, 1H, ^4^ *J* = 1.0, ^5^ *J* = 2.0, H^2^), 7.01 (*d*, 1H, ^5^ *J* = 5.0, H^4^), 7.43 (*dd*, 1H, ^2^ *J* = 3.0, ^4^ *J* = 4.5, H^5^), 3.32 (*s*, 2H, H^6^), 9.14 (*s*, 1H, H^8^), 4.19 (*s*, 2H, H^9^). ^13^C NMR [Bruker XL-500, 125 MHz, *d*_6_-DMSO, δ (ppm)]: 122.06 (C^2^), 135.95 (C^3^),128.62 (C^4^), 125.59 (C^5^), 35.10 (C^7^), 169.17 (C^8^). Calculation for C_6_H_8_O_2_N_2_S: *M* = 172 au.

### Synthesis of 2-(thio­phen-3-yl)acetohydrazide, 2   

Methyl thio­phene-2-acetate, **1** (5 mmol), was added to an excess of hydrazine hydrate (40 mmol) in ethanol (20 ml). The mixture was refluxed for 6 h. The reaction mixture was allowed to cool. The resulting precipitate was filtered and recrystallized from ethanol solution to give 0.57 g (yield 74%) of hydrazide **2** in the form of white crystals (m.p. 343 K). IR (Nicolet Impact 410 FTIR, KBr, cm^−1^): 3323, 3068 (ν_NH_), 3068, 2957 (ν_CH_), 1641 (ν_C=O_), 1526 (ν_C=C_ thio­phene). ^1^H NMR [Bruker XL-500, 500 MHz, *d*_6_-DMSO, δ (ppm), *J* (Hz)]: 7.22 (*dd*, 1H, ^4^ *J* = 1.0, ^5^ *J* = 2.0, H^2^), 7.01 (*d*, 1H, ^5^ *J* = 5.0, H^4^), 7.43 (*dd*, 1H, ^2^ *J* = 3.0, ^4^ *J* = 4.5, H^5^), 3.32 (*s*, 2H, H^6^), 9.14 (*s*, 1H, H^8^), 4.19 (*s*, 2H, H^9^). ^13^C NMR [Bruker XL-500, 125 MHz, *d*_6_-DMSO, δ (ppm)]: 122.06 (C^2^), 135.95 (C^3^),128.62 (C^4^), 125.59 (C^5^), 35.10 (C^7^), 169.17 (C^8^). Calculation for C_6_H_8_O_2_N_2_S: *M* = 172 au.

### Synthesis of *N*-(4-oxo-2-sulfanyl­idene-1,3-thia­zolidin-3-yl)-2-(thio­phen-3-yl)acetamide, 3   

A mixture of hydrazide **2** (10 mmol) and thio­carbonyl­bis­thio­glycolic acid (10 mmol) in ethanol (5 ml) was refluxed for 8 h. After cooling, the resulting precipitate was filtered off, dried and recrystallized from ethanol solution to give 1.66 g (yield 61%) of **3** as a pale-yellow crystals (m.p. 372 K). IR (Nicolet Impact 410 FTIR, KBr, cm^−1^): 3442, 3292, 3226 (ν_NH_), 3148, 2965, 2921 (ν_CH_), 1727,1684 (ν_C=O_), 1614, 1532 (ν_C=C_ thio­phene), 1244, 1177 (ν_C=S_). Calculation for C_9_H_8_O_2_N_2_S_3_: *M* = 272 au.

## Refinement   

Crystal data, data collection and structure refinement details are summarized in Table 2 ▸. Both thio­phene rings are disordered over two positions by a rotation of approximately 180° around the C5—C3 or C15—C13 bond for mol­ecules *A* and *B*, respectively. The final occupancy factors are 0.6727 (17) and 0.3273 (17) for mol­ecule *A*, and 0.7916 (19) and 0.2084 (19) for mol­ecule *B*. Bond lengths and angles in the disordered thio­phene rings were restrained to target values derived from mean values observed in 3-CH_2_-thio­phene fragments in the CSD (Groom *et al.*, 2016 ▸). The same anisotropic displacement parameters were used for equivalent atoms in the disordered thio­phene rings (*e.g.* EADP C1*A* C1*B*). The H atoms attached to atoms N1 and N11 were found in the difference density Fourier map and refined freely. The other H atoms were placed in idealized positions and refined in riding mode, with *U*_iso_(H) values assigned as 1.2*U*_eq_ of the parent atoms, with C—H distances of 0.95 (aromatic) and 0.99 Å (CH_2_). In the final cycles of refinement, four outliers were omitted.

## Supplementary Material

Crystal structure: contains datablock(s) I. DOI: 10.1107/S2056989017007629/hb7673sup1.cif

Structure factors: contains datablock(s) I. DOI: 10.1107/S2056989017007629/hb7673Isup2.hkl

Supporting information file. DOI: 10.1107/S2056989017007629/hb7673Isup3.cml

CCDC reference: 1551679

Additional supporting information:  crystallographic information; 3D view; checkCIF report

## Figures and Tables

**Table 1:** Hydrogen-bond geometry (Å, °) *Cg*1 and *Cg*2 are the centroids of the S1*A*/C1*A*–C4*A* and S11*A*/C11*A*–C14*A* rings, respectively.

*D*—H⋯*A*	*D*—H	H⋯*A*	*D*⋯*A*	*D*—H⋯*A*
N1—H1⋯O11	0.824 (19)	1.973 (19)	2.7923 (16)	173.1 (18)
N11—H11⋯O1^i^	0.819 (19)	2.189 (19)	2.8436 (16)	137.1 (16)
N11—H11⋯O2^ii^	0.819 (19)	2.519 (18)	3.0965 (16)	128.6 (15)
C5—H5*A*⋯O12^iii^	0.99	2.46	3.3901 (19)	156
C9—H9*A*⋯O2^iv^	0.99	2.53	3.2443 (19)	129
C9—H9*B*⋯S13^ii^	0.99	2.81	3.6570 (17)	144
C15—H15*A*⋯O2^ii^	0.99	2.37	3.2862 (19)	154
C9—H9*A*⋯*Cg*1^iv^	0.99	2.73	3.276 (3)	115
C19—H19*A*⋯*Cg*2^iii^	0.99	2.77	3.480 (2)	129

Symmetry codes: (i) 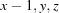; (ii) 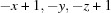; (iii) 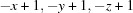; (iv) 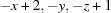.

**Table 2:** Experimental details

Crystal data	
Chemical formula	C_9_H_8_N_2_O_2_S_3_
*M*_r_	272.35
Crystal system, space group	Triclinic, *P* 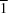
Temperature (K)	100
*a*, *b*, *c* (Å)	9.6205 (3), 10.8252 (3), 11.5073 (3)
α, β, γ (°)	97.836 (2), 102.720 (2), 95.047 (2)
*V* (Å^3^)	1149.42 (6)
*Z*	4
Radiation type	Mo *K*α
μ (mm^−1^)	0.63
Crystal size (mm)	0.22 × 0.07 × 0.04

Data collection	
Diffractometer	Bruker APEXII CCD
Absorption correction	Multi-scan (*SADABS*; Bruker, 2014 ▸)
*T*_min_, *T*_max_	0.691, 0.746
No. of measured, independent and observed [*I* > 2σ(*I*)] reflections	37647, 6098, 4985
*R*_int_	0.042
(sin θ/λ)_max_ (Å^−1^)	0.682

Refinement	
*R*[*F* ^2^ > 2σ(*F* ^2^)], *wR*(*F* ^2^), *S*	0.032, 0.077, 1.03
No. of reflections	6098
No. of parameters	323
No. of restraints	40
H-atom treatment	H atoms treated by a mixture of independent and constrained refinement
Δρ_max_, Δρ_min_ (e Å^−3^)	0.38, −0.25

Computer programs: *APEX2* (Bruker, 2014 ▸), *SAINT* (Bruker, 2013 ▸), *SHELXS1997* (Sheldrick, 2008 ▸), *SHELXL* (Sheldrick, 2015 ▸) and *OLEX2* (Dolomanov *et al.*, 2009 ▸).

## supplementary crystallographic information

### Crystal data

**Table:**

C_9_H_8_N_2_O_2_S_3_	*Z* = 4
*M**_r_* = 272.35	*F*(000) = 560
Triclinic, *P*1	*D*_x_ = 1.574 Mg m^−^^3^
*a* = 9.6205 (3) Å	Mo *K*α radiation, λ = 0.71073 Å
*b* = 10.8252 (3) Å	Cell parameters from 9925 reflections
*c* = 11.5073 (3) Å	θ = 3.1–30.6°
α = 97.836 (2)°	µ = 0.63 mm^−^^1^
β = 102.720 (2)°	*T* = 100 K
γ = 95.047 (2)°	Block, colourless
*V* = 1149.42 (6) Å^3^	0.22 × 0.07 × 0.04 mm

### Data collection

**Table:**

Bruker APEX-II CCD diffractometer	4985 reflections with *I* > 2σ(*I*)
φ and ω scans	*R*_int_ = 0.042
Absorption correction: multi-scan (SADABS; Bruker, 2014)	θ_max_ = 29.0°, θ_min_ = 2.9°
*T*_min_ = 0.691, *T*_max_ = 0.746	*h* = −13→13
37647 measured reflections	*k* = −14→14
6098 independent reflections	*l* = −15→15

### Refinement

**Table:**

Refinement on *F*^2^	Primary atom site location: structure-invariant direct methods
Least-squares matrix: full	Hydrogen site location: mixed
*R*[*F*^2^ > 2σ(*F*^2^)] = 0.032	H atoms treated by a mixture of independent and constrained refinement
*wR*(*F*^2^) = 0.077	*w* = 1/[σ^2^(*F*_o_^2^) + (0.0367*P*)^2^ + 0.3868*P*] where *P* = (*F*_o_^2^ + 2*F*_c_^2^)/3
*S* = 1.03	(Δ/σ)_max_ = 0.001
6098 reflections	Δρ_max_ = 0.38 e Å^−^^3^
323 parameters	Δρ_min_ = −0.25 e Å^−^^3^
40 restraints	

### Special details

**Table:**

Geometry. All esds (except the esd in the dihedral angle between two l.s. planes) are estimated using the full covariance matrix. The cell esds are taken into account individually in the estimation of esds in distances, angles and torsion angles; correlations between esds in cell parameters are only used when they are defined by crystal symmetry. An approximate (isotropic) treatment of cell esds is used for estimating esds involving l.s. planes.

### Fractional atomic coordinates and isotropic or equivalent isotropic displacement parameters (Å^2^)

**Table:**

	*x*	*y*	*z*	*U*_iso_*/*U*_eq_	Occ. (<1)
C1A	0.7012 (6)	0.0875 (6)	0.1304 (5)	0.0435 (14)	0.6727 (17)
H1A	0.627681	0.019090	0.098279	0.052*	0.6727 (17)
C1B	0.8309 (14)	0.1396 (12)	0.0779 (11)	0.0435 (14)	0.3273 (17)
H1B	0.865405	0.109102	0.009449	0.052*	0.3273 (17)
C2A	0.7136 (6)	0.1688 (5)	0.2354 (6)	0.0292 (11)	0.6727 (17)
H2A	0.648572	0.161127	0.286104	0.035*	0.6727 (17)
C2B	0.8979 (13)	0.2418 (14)	0.1659 (11)	0.0292 (11)	0.3273 (17)
H2B	0.982111	0.291811	0.161213	0.035*	0.3273 (17)
C3	0.83043 (16)	0.26481 (14)	0.26249 (13)	0.0229 (3)	
C4A	0.9131 (5)	0.2528 (5)	0.1785 (4)	0.0196 (7)	0.6727 (17)
H4A	0.998639	0.306140	0.183286	0.024*	0.6727 (17)
C4B	0.7015 (13)	0.1835 (11)	0.2382 (11)	0.0196 (7)	0.3273 (17)
H4B	0.634214	0.186979	0.287497	0.024*	0.3273 (17)
C5	0.87295 (17)	0.36183 (14)	0.37536 (13)	0.0244 (3)	
H5A	0.786386	0.394860	0.394322	0.029*	
H5B	0.937159	0.432803	0.362651	0.029*	
C6	0.94939 (15)	0.30218 (12)	0.47922 (12)	0.0195 (3)	
C7	0.99694 (15)	0.26354 (14)	0.76650 (13)	0.0219 (3)	
C8	0.91801 (15)	0.08293 (13)	0.61312 (13)	0.0211 (3)	
C9	0.99968 (17)	0.02374 (14)	0.71264 (13)	0.0255 (3)	
H9A	1.082011	−0.012463	0.688482	0.031*	
H9B	0.936797	−0.044373	0.731533	0.031*	
N1	0.87242 (13)	0.28734 (11)	0.56472 (11)	0.0205 (2)	
H1	0.784 (2)	0.2785 (16)	0.5452 (15)	0.023 (4)*	
N2	0.92535 (12)	0.21256 (11)	0.64903 (10)	0.0200 (2)	
O1	1.06854 (11)	0.26997 (10)	0.48825 (10)	0.0263 (2)	
O2	0.85315 (11)	0.03015 (10)	0.51434 (10)	0.0263 (2)	
S1A	0.84226 (13)	0.12988 (10)	0.06504 (10)	0.0260 (2)	0.6727 (17)
S1B	0.6811 (3)	0.0788 (3)	0.1107 (3)	0.0260 (2)	0.3273 (17)
S2	1.06254 (4)	0.14561 (4)	0.84299 (3)	0.02640 (9)	
S3	1.01734 (5)	0.41087 (4)	0.82790 (4)	0.03324 (10)	
C11A	0.4107 (4)	0.3665 (5)	0.8615 (4)	0.0348 (9)	0.7916 (19)
H11A	0.353131	0.424722	0.890676	0.042*	0.7916 (19)
C12A	0.3757 (4)	0.2893 (6)	0.7510 (5)	0.0260 (8)	0.7916 (19)
H12A	0.287724	0.288656	0.693747	0.031*	0.7916 (19)
C11B	0.6014 (12)	0.3318 (14)	0.9163 (10)	0.0348 (9)	0.2084 (19)
H11B	0.675966	0.365869	0.985263	0.042*	0.2084 (19)
C12B	0.6096 (19)	0.238 (3)	0.819 (2)	0.0260 (8)	0.2084 (19)
H12B	0.693246	0.198134	0.815566	0.031*	0.2084 (19)
C13	0.48263 (16)	0.20962 (13)	0.72904 (12)	0.0209 (3)	
C14A	0.5992 (5)	0.2286 (6)	0.8247 (5)	0.0279 (11)	0.7916 (19)
H14A	0.680976	0.184997	0.826702	0.033*	0.7916 (19)
C14B	0.3780 (19)	0.274 (3)	0.7503 (19)	0.0279 (11)	0.2084 (19)
H14B	0.285834	0.266998	0.697131	0.033*	0.2084 (19)
C15	0.46840 (16)	0.11790 (13)	0.61472 (12)	0.0216 (3)	
H15A	0.378555	0.059746	0.598778	0.026*	
H15B	0.550170	0.067657	0.623555	0.026*	
C16	0.46640 (14)	0.19004 (12)	0.51108 (12)	0.0173 (3)	
C17	0.31832 (14)	0.24127 (13)	0.24248 (12)	0.0192 (3)	
C18	0.33768 (14)	0.41284 (13)	0.40333 (13)	0.0190 (3)	
C19	0.33812 (17)	0.48314 (13)	0.29957 (13)	0.0247 (3)	
H19A	0.428495	0.540567	0.315257	0.030*	
H19B	0.256618	0.533625	0.288467	0.030*	
N11	0.33329 (13)	0.20086 (11)	0.44504 (10)	0.0173 (2)	
H11	0.261 (2)	0.1832 (16)	0.4689 (15)	0.025 (4)*	
N12	0.32383 (12)	0.28276 (10)	0.36245 (10)	0.0168 (2)	
O11	0.57399 (10)	0.23773 (10)	0.48702 (9)	0.0240 (2)	
O12	0.34760 (11)	0.45633 (9)	0.50671 (9)	0.0247 (2)	
S11A	0.58246 (9)	0.33791 (8)	0.93937 (6)	0.0371 (2)	0.7916 (19)
S11B	0.4286 (6)	0.3711 (5)	0.8833 (4)	0.0371 (2)	0.2084 (19)
S12	0.32221 (5)	0.36801 (4)	0.16587 (3)	0.02777 (9)	
S13	0.30889 (4)	0.09510 (3)	0.17993 (3)	0.02742 (9)	

### Atomic displacement parameters (Å^2^)

**Table:**

	*U*^11^	*U*^22^	*U*^33^	*U*^12^	*U*^13^	*U*^23^
C1A	0.037 (2)	0.057 (3)	0.038 (3)	0.0079 (19)	0.0078 (18)	0.012 (2)
C1B	0.037 (2)	0.057 (3)	0.038 (3)	0.0079 (19)	0.0078 (18)	0.012 (2)
C2A	0.0221 (16)	0.040 (3)	0.0266 (15)	0.0060 (14)	0.0066 (12)	0.0074 (15)
C2B	0.0221 (16)	0.040 (3)	0.0266 (15)	0.0060 (14)	0.0066 (12)	0.0074 (15)
C3	0.0264 (7)	0.0246 (7)	0.0184 (7)	0.0087 (6)	0.0044 (5)	0.0041 (5)
C4A	0.0234 (17)	0.0195 (14)	0.0152 (15)	0.0055 (11)	0.0024 (12)	0.0019 (10)
C4B	0.0234 (17)	0.0195 (14)	0.0152 (15)	0.0055 (11)	0.0024 (12)	0.0019 (10)
C5	0.0303 (8)	0.0230 (7)	0.0212 (7)	0.0101 (6)	0.0067 (6)	0.0028 (5)
C6	0.0185 (7)	0.0173 (6)	0.0204 (7)	0.0017 (5)	0.0028 (5)	−0.0019 (5)
C7	0.0181 (7)	0.0271 (7)	0.0210 (7)	0.0045 (6)	0.0049 (5)	0.0035 (5)
C8	0.0162 (6)	0.0232 (7)	0.0246 (7)	0.0002 (5)	0.0080 (5)	0.0026 (5)
C9	0.0300 (8)	0.0213 (7)	0.0251 (7)	0.0012 (6)	0.0061 (6)	0.0049 (6)
N1	0.0147 (6)	0.0262 (6)	0.0206 (6)	0.0072 (5)	0.0024 (5)	0.0033 (5)
N2	0.0177 (6)	0.0224 (6)	0.0195 (6)	0.0038 (5)	0.0034 (4)	0.0027 (4)
O1	0.0170 (5)	0.0314 (6)	0.0313 (6)	0.0057 (4)	0.0068 (4)	0.0042 (4)
O2	0.0220 (5)	0.0260 (5)	0.0268 (5)	−0.0008 (4)	0.0025 (4)	−0.0017 (4)
S1A	0.0297 (4)	0.0297 (4)	0.0182 (3)	0.0060 (3)	0.0044 (2)	0.0029 (2)
S1B	0.0297 (4)	0.0297 (4)	0.0182 (3)	0.0060 (3)	0.0044 (2)	0.0029 (2)
S2	0.0324 (2)	0.02659 (19)	0.02008 (18)	0.00490 (15)	0.00387 (14)	0.00602 (14)
S3	0.0405 (2)	0.02536 (19)	0.0278 (2)	0.01014 (17)	−0.00280 (17)	−0.00329 (15)
C11A	0.0280 (16)	0.0542 (18)	0.029 (2)	0.0119 (13)	0.0103 (14)	0.0206 (16)
C12A	0.0257 (11)	0.029 (2)	0.0267 (10)	0.0055 (10)	0.0130 (9)	0.0047 (11)
C11B	0.0280 (16)	0.0542 (18)	0.029 (2)	0.0119 (13)	0.0103 (14)	0.0206 (16)
C12B	0.0257 (11)	0.029 (2)	0.0267 (10)	0.0055 (10)	0.0130 (9)	0.0047 (11)
C13	0.0285 (7)	0.0181 (6)	0.0170 (6)	0.0015 (5)	0.0070 (5)	0.0039 (5)
C14A	0.0390 (17)	0.0221 (15)	0.0190 (12)	0.0073 (14)	−0.0020 (12)	0.0030 (11)
C14B	0.0390 (17)	0.0221 (15)	0.0190 (12)	0.0073 (14)	−0.0020 (12)	0.0030 (11)
C15	0.0268 (7)	0.0170 (6)	0.0185 (7)	0.0021 (5)	0.0009 (5)	0.0018 (5)
C16	0.0194 (6)	0.0150 (6)	0.0161 (6)	0.0041 (5)	0.0032 (5)	−0.0022 (5)
C17	0.0172 (6)	0.0217 (7)	0.0184 (6)	0.0033 (5)	0.0042 (5)	0.0017 (5)
C18	0.0144 (6)	0.0186 (6)	0.0237 (7)	0.0043 (5)	0.0048 (5)	0.0004 (5)
C19	0.0307 (8)	0.0192 (7)	0.0242 (7)	0.0064 (6)	0.0048 (6)	0.0041 (5)
N11	0.0161 (6)	0.0194 (6)	0.0174 (5)	0.0016 (4)	0.0053 (4)	0.0044 (4)
N12	0.0178 (5)	0.0164 (5)	0.0162 (5)	0.0035 (4)	0.0042 (4)	0.0016 (4)
O11	0.0159 (5)	0.0321 (6)	0.0250 (5)	0.0045 (4)	0.0059 (4)	0.0054 (4)
O12	0.0264 (5)	0.0228 (5)	0.0248 (5)	0.0022 (4)	0.0098 (4)	−0.0029 (4)
S11A	0.0573 (5)	0.0334 (3)	0.0176 (3)	0.0122 (3)	0.0034 (2)	−0.0020 (2)
S11B	0.0573 (5)	0.0334 (3)	0.0176 (3)	0.0122 (3)	0.0034 (2)	−0.0020 (2)
S12	0.0389 (2)	0.02616 (19)	0.01882 (18)	0.00436 (16)	0.00618 (15)	0.00631 (14)
S13	0.0352 (2)	0.02192 (18)	0.02263 (18)	0.00242 (15)	0.00702 (15)	−0.00483 (13)

### Geometric parameters (Å, º)

**Table:**

C1A—H1A	0.9500	C5—C6	1.514 (2)
C1B—H1B	0.9500	C6—N1	1.3730 (18)
C1A—C2A	1.370 (8)	C6—O1	1.2141 (17)
C2A—H2A	0.9500	C7—N2	1.3878 (18)
C1B—C2B	1.395 (13)	C7—S2	1.7339 (15)
C2B—H2B	0.9500	C7—S3	1.6303 (15)
C2B—C3	1.410 (11)	C8—C9	1.494 (2)
C2A—C3	1.412 (6)	C8—N2	1.3988 (18)
C4A—H4A	0.9500	C8—O2	1.2065 (17)
C4B—H4B	0.9500	C9—H9A	0.9900
C4A—S1A	1.707 (4)	C9—H9B	0.9900
C1A—S1A	1.747 (7)	C9—S2	1.8102 (15)
C4B—S1B	1.690 (11)	N1—H1	0.825 (18)
C1B—S1B	1.672 (11)	N1—N2	1.3874 (16)
C11A—H11A	0.9500	C13—C14A	1.366 (4)
C11A—C12A	1.378 (6)	C13—C14B	1.323 (14)
C12A—H12A	0.9500	C13—C15	1.5086 (18)
C11B—H11B	0.9500	C15—H15A	0.9900
C11B—C12B	1.430 (16)	C15—H15B	0.9900
C12B—H12B	0.9500	C15—C16	1.5096 (19)
C12A—C13	1.442 (4)	C16—N11	1.3613 (17)
C12B—C13	1.395 (15)	C16—O11	1.2190 (17)
C14A—H14A	0.9500	C17—N12	1.3802 (17)
C14B—H14B	0.9500	C17—S12	1.7310 (14)
C11A—S11A	1.773 (4)	C17—S13	1.6326 (14)
C14A—S11A	1.694 (4)	C18—C19	1.501 (2)
C14B—S11B	1.680 (15)	C18—N12	1.4073 (17)
C11B—S11B	1.726 (12)	C18—O12	1.1982 (16)
C3—C4A	1.380 (5)	C19—H19A	0.9900
C3—C4B	1.407 (12)	C19—H19B	0.9900
C3—C5	1.509 (2)	C19—S12	1.8121 (15)
C5—H5A	0.9900	N11—H11	0.819 (18)
C5—H5B	0.9900	N11—N12	1.3797 (16)
			
C4A—S1A—C1A	92.8 (2)	C8—C9—H9B	110.3
S1A—C1A—H1A	125.5	C8—C9—S2	107.29 (10)
C2A—C1A—H1A	125.5	H9A—C9—H9B	108.5
S1B—C1B—H1B	125.0	S2—C9—H9A	110.3
C2B—C1B—H1B	125.0	S2—C9—H9B	110.3
C1A—C2A—H2A	122.6	C6—N1—H1	119.8 (12)
C1B—C2B—H2B	122.7	C6—N1—N2	116.15 (11)
C1A—C2A—C3	114.8 (4)	N2—N1—H1	112.4 (12)
C1B—C2B—C3	114.5 (8)	C7—N2—C8	118.28 (12)
S1A—C4A—H4A	124.2	N1—N2—C7	121.89 (12)
C1B—S1B—C4B	94.1 (5)	N1—N2—C8	119.59 (11)
S1B—C4B—H4B	124.1	C7—S2—C9	93.85 (7)
C2A—C1A—S1A	109.0 (4)	C14B—C13—C15	122.9 (6)
C2B—C1B—S1B	109.9 (7)	C13—C15—H15A	109.9
C14A—S11A—C11A	93.1 (2)	C3—C2A—H2A	122.6
C12A—C11A—H11A	126.1	C3—C2B—H2B	122.7
S11A—C11A—H11A	126.1	C3—C4A—H4A	124.2
C11A—C12A—H12A	122.6	C3—C4B—H4B	124.1
C14B—S11B—C11B	94.8 (6)	C13—C15—H15B	109.9
S11B—C11B—H11B	127.0	C3—C4A—S1A	111.5 (2)
C12B—C11B—H11B	127.0	C3—C4B—S1B	111.7 (6)
C11B—C12B—H12B	123.3	C13—C15—C16	108.99 (11)
C11A—C12A—C13	114.9 (3)	H15A—C15—H15B	108.3
S11A—C14A—H14A	123.6	C16—C15—H15A	109.9
S11B—C14B—H14B	124.3	C16—C15—H15B	109.9
C12A—C11A—S11A	107.8 (3)	N11—C16—C15	115.08 (12)
C12B—C11B—S11B	106.0 (8)	O11—C16—C15	123.93 (12)
C4A—C3—C2A	111.8 (2)	O11—C16—N11	120.98 (12)
C4B—C3—C2B	109.4 (6)	N12—C17—S12	109.87 (10)
C4A—C3—C5	122.36 (18)	N12—C17—S13	125.87 (11)
C4B—C3—C5	120.4 (4)	S13—C17—S12	124.26 (8)
C2A—C3—C5	125.5 (2)	N12—C18—C19	109.81 (11)
C2B—C3—C5	130.2 (4)	O12—C18—C19	127.34 (13)
C3—C5—H5A	109.8	O12—C18—N12	122.85 (13)
C3—C5—H5B	109.8	C18—C19—H19A	110.2
C14A—C13—C12A	111.3 (3)	C18—C19—H19B	110.2
C14B—C13—C12B	114.2 (8)	C18—C19—S12	107.46 (10)
C14A—C13—C15	124.3 (2)	H19A—C19—H19B	108.5
C12B—C13—C15	122.8 (6)	S12—C19—H19A	110.2
C12A—C13—C15	124.4 (2)	S12—C19—H19B	110.2
C3—C5—C6	109.31 (11)	C16—N11—H11	121.5 (12)
H5A—C5—H5B	108.3	C16—N11—N12	117.50 (11)
C6—C5—H5A	109.8	N12—N11—H11	117.0 (12)
C6—C5—H5B	109.8	C17—N12—C18	118.85 (11)
N1—C6—C5	114.18 (12)	N11—N12—C17	121.30 (11)
O1—C6—C5	123.98 (13)	N11—N12—C18	119.39 (11)
O1—C6—N1	121.84 (13)	C17—S12—C19	93.94 (7)
N2—C7—S2	109.78 (10)	C13—C12A—H12A	122.6
N2—C7—S3	126.68 (11)	C13—C12B—C11B	113.4 (10)
S3—C7—S2	123.53 (9)	C13—C12B—H12B	123.3
N2—C8—C9	110.62 (12)	C13—C14A—H14A	123.6
O2—C8—C9	126.72 (14)	C13—C14B—H14B	124.3
O2—C8—N2	122.66 (14)	C13—C14A—S11A	112.9 (3)
C8—C9—H9A	110.3	C13—C14B—S11B	111.5 (9)
			
C2B—C1B—S1B—C4B	0.0 (13)	N2—C8—C9—S2	4.36 (14)
C12A—C11A—S11A—C14A	0.2 (6)	O1—C6—N1—N2	−11.93 (19)
C2A—C1A—S1A—C4A	0.2 (5)	O2—C8—C9—S2	−175.56 (12)
C12B—C11B—S11B—C14B	−2 (2)	O2—C8—N2—C7	176.99 (13)
S1B—C1B—C2B—C3	3.4 (18)	O2—C8—N2—N1	−8.4 (2)
S1A—C1A—C2A—C3	1.4 (6)	S2—C7—N2—C8	−0.09 (15)
S11B—C11B—C12B—C13	2 (3)	S2—C7—N2—N1	−174.54 (10)
S11A—C11A—C12A—C13	0.0 (7)	S3—C7—N2—C8	−179.36 (11)
C1B—C2B—C3—C4B	−5.7 (17)	S3—C7—N2—N1	6.2 (2)
C1A—C2A—C3—C4A	−2.9 (7)	S3—C7—S2—C9	−178.31 (10)
C1A—C2A—C3—C5	−176.5 (4)	C12A—C13—C15—C16	65.3 (4)
C1B—C2B—C3—C5	177.4 (9)	C12B—C13—C15—C16	−106.2 (18)
C2A—C3—C5—C6	76.8 (3)	C14A—C13—C15—C16	−114.1 (4)
C4B—C3—C5—C6	84.8 (6)	C14B—C13—C15—C16	70.8 (19)
C4A—C3—C5—C6	−96.2 (4)	C13—C14B—S11B—C11B	2 (2)
C11B—C12B—C13—C14B	0 (3)	C13—C14A—S11A—C11A	−0.4 (5)
C11A—C12A—C13—C14A	−0.3 (6)	C13—C15—C16—N11	−96.18 (14)
C11A—C12A—C13—C15	−179.8 (4)	C13—C15—C16—O11	82.80 (16)
C11B—C12B—C13—C15	176.9 (15)	C15—C13—C14B—S11B	−178.4 (10)
C2A—C3—C4A—S1A	2.9 (6)	C15—C13—C14A—S11A	179.9 (2)
C2B—C3—C4B—S1B	5.5 (12)	C15—C16—N11—N12	169.47 (11)
C2B—C3—C5—C6	−98.6 (10)	C16—N11—N12—C17	95.69 (15)
C12A—C13—C14A—S11A	0.4 (5)	C16—N11—N12—C18	−76.45 (15)
C12B—C13—C14B—S11B	−1 (3)	C18—C19—S12—C17	−0.76 (11)
C3—C4A—S1A—C1A	−1.8 (4)	C19—C18—N12—C17	2.19 (17)
C3—C4B—S1B—C1B	−3.3 (10)	C19—C18—N12—N11	174.53 (11)
C3—C5—C6—N1	−110.03 (14)	N12—C17—S12—C19	1.91 (11)
C3—C5—C6—O1	70.25 (18)	N12—C18—C19—S12	−0.57 (14)
C5—C3—C4B—S1B	−177.2 (4)	O11—C16—N11—N12	−9.54 (18)
C5—C3—C4A—S1A	176.8 (2)	O12—C18—C19—S12	179.28 (12)
C5—C6—N1—N2	168.34 (12)	O12—C18—N12—C17	−177.67 (13)
C6—N1—N2—C7	104.36 (15)	O12—C18—N12—N11	−5.33 (19)
C6—N1—N2—C8	−70.01 (16)	S12—C17—N12—C18	−2.78 (15)
C8—C9—S2—C7	−3.86 (11)	S12—C17—N12—N11	−174.96 (10)
C9—C8—N2—C7	−2.94 (17)	S13—C17—N12—C18	177.43 (10)
C9—C8—N2—N1	171.64 (12)	S13—C17—N12—N11	5.25 (19)
N2—C7—S2—C9	2.39 (11)	S13—C17—S12—C19	−178.29 (10)

### Hydrogen-bond geometry (Å, º)

*Cg*1 and *Cg*2 are the centroids of the S1A/C1A–C4A and S11A/C11A–C14A rings, respectively.

**Table:**

*D*—H···*A*	*D*—H	H···*A*	*D*···*A*	*D*—H···*A*
N1—H1···O11	0.824 (19)	1.973 (19)	2.7923 (16)	173.1 (18)
N11—H11···O1^i^	0.819 (19)	2.189 (19)	2.8436 (16)	137.1 (16)
N11—H11···O2^ii^	0.819 (19)	2.519 (18)	3.0965 (16)	128.6 (15)
C5—H5*A*···O12^iii^	0.99	2.46	3.3901 (19)	156
C9—H9*A*···O2^iv^	0.99	2.53	3.2443 (19)	129
C9—H9*B*···S13^ii^	0.99	2.81	3.6570 (17)	144
C15—H15*A*···O2^ii^	0.99	2.37	3.2862 (19)	154
C9—H9*A*···*Cg*1^iv^	0.99	2.73	3.276 (3)	115
C19—H19*A*···*Cg*2^iii^	0.99	2.77	3.480 (2)	129

Symmetry codes: (i) *x*−1, *y*, *z*; (ii) −*x*+1, −*y*, −*z*+1; (iii) −*x*+1, −*y*+1, −*z*+1; (iv) −*x*+2, −*y*, −*z*+1.
